# Noninvasive respiratory support for COVID-19 patients: when, for whom, and how?

**DOI:** 10.1186/s40560-021-00593-1

**Published:** 2022-01-15

**Authors:** Zachary P. Sullivan, Luca Zazzeron, Lorenzo Berra, Dean R. Hess, Edward A. Bittner, Marvin G. Chang

**Affiliations:** Department of Anesthesia, Critical Care and Pain Medicine, Massachusetts General Hospital, MA Boston, USA

**Keywords:** Noninvasive respiratory support (NIRS), High flow nasal cannula (HFNC), Continuous positive airway pressure (CPAP), Noninvasive ventilation (NIV), COVID-19, Invasive mechanical ventilation (IMV), SARS, MERS, H1N1, Hypoxemic respiratory failure, Acute respiratory failure (ARF), Acute respiratory distress syndrome (ARDS)

## Abstract

The significant mortality rate and prolonged ventilator days associated with invasive mechanical ventilation (IMV) in patients with severe COVID-19 have incited a debate surrounding the use of noninvasive respiratory support (NIRS) (i.e., HFNC, CPAP, NIV) as a potential treatment strategy. Central to this debate is the role of NIRS in preventing intubation in patients with mild respiratory disease and the potential beneficial effects on both patient outcome and resource utilization. However, there remains valid concern that use of NIRS may prolong time to intubation and lung protective ventilation in patients with more advanced disease, thereby worsening respiratory mechanics via self-inflicted lung injury. In addition, the risk of aerosolization with the use of NIRS has the potential to increase healthcare worker (HCW) exposure to the virus. We review the existing literature with a focus on rationale, patient selection and outcomes associated with the use of NIRS in COVID-19 and prior pandemics, as well as in patients with acute respiratory failure due to different etiologies (i.e., COPD, cardiogenic pulmonary edema, etc.) to understand the potential role of NIRS in COVID-19 patients. Based on this analysis we suggest an algorithm for NIRS in COVID-19 patients which includes indications and contraindications for use, monitoring recommendations, systems-based practices to reduce HCW exposure, and predictors of NIRS failure. We also discuss future research priorities for addressing unanswered questions regarding NIRS use in COVID-19 with the goal of improving patient outcomes.

**Supplementary Information:**

The online version contains supplementary material available at 10.1186/s40560-021-00593-1.

## Introduction

The high mortality rate and prolonged ventilator days associated with invasive mechanical ventilation (IMV) reported in COVID-19 patients have renewed the discussion surrounding the utility of noninvasive respiratory support (NIRS), an umbrella term encompassing high flow nasal cannula (HFNC), continuous positive airway pressure (CPAP), and noninvasive ventilation (NIV). NIV has been shown to be efficacious in critically ill patients with conditions, such as chronic obstructive pulmonary disease (COPD), cardiogenic pulmonary edema, obstructive sleep apnea (OSA), and hypercapneic respiratory failure, whereas its utility is less clear in the management of patients with pneumonia, acute respiratory distress syndrome (ARDS), and particularly, COVID-19. Knowledge gained from the use of NIRS in prior viral pandemics such as SARS, MERS, and H1N1 may provide insight into appropriate use during the COVID-19 pandemic. Here, we outline the three most commonly utilized forms of NIRS, discuss the evolving literature pertaining to NIRS with and without proning, outline the aerosolization risks with different forms of NIRS, and review available evidence for the use of NIRS in patients with COVID-19 as well as in viral pneumonia from prior pandemics, ARDS, and community acquired pneumonia (CAP). Based on this analysis we suggest an algorithm for NIRS in COVID-19 patients which includes indications and contraindications for use, monitoring recommendations, practices to reduce HCW exposure, and predictors of NIRS failure. Finally, we discuss research priorities for addressing unanswered questions regarding the use of NIRS in COVID-19.

## Noninvasive respiratory support strategies

Additional file 1: Text S1 outlines the most clinically used NIRS strategies (HFNC, CPAP, NIV), the risks and benefits of each, and the different delivery methods available, as well as a discussion of the ROX index which has been validated as a tool to assess the likelihood of HFNC failure in patients with acute hypoxemic respiratory failure.

### Modes of transmission and risk of aerosolization

COVID-19 has various modes of transmission. While contact and fomite transmission do occur, the primary modes of transmission appear to be droplet and airborne. Airborne transmission is a direct result of inhalation of aerosolized viral particles. The primary differentiating factor between droplets and aerosols is size. According to the World Health Organization, respiratory droplets are greater than 5–10 µm in diameter, whereas aerosols are less than 5 µm in diameter. Droplet transmission occurs within minutes of exposure to an infected individual and is typically the result of droplet formation from talking, singing, coughing, sneezing, or laughing. Due to the size and weight of droplet particles, they do not stay suspended in air for very long, and transmission typically occurs within six feet of the infected individual. In contrast, airborne transmission is a result of aerosolized particles remaining suspended in the air for a prolonged period of time, which makes this mode of transmission particularly concerning in large crowds, indoors, or in areas with poor ventilation. Of particular concern to healthcare workers is the role of aerosol generating procedures in the spread of COVID-19. Aerosol generating procedures such as laryngoscopy and bronchoscopy are thought to produce high concentrations of aerosolized particles which would increase the risk of airborne spread. While evidence remains lacking as to the precise proportion of spread that is due to airborne transmission from aerosolized particles, healthcare workers must remain vigilant when performing aerosol generating procedures to prevent inadvertent spread of COVID-19.

Much of our understanding of aerosolization associated with different ventilatory support strategies arose from research catalyzed by the previous pandemics of the twenty-first century. Yu et al. found six risk factors responsible for nosocomial spread of SARS which included minimum distance between beds (≤ 1 m), access to washing/changing facilities for staff, resuscitation administered on the ward, symptomatic staff continuing to work, and whether the patient required supplemental oxygen or NIV [1]. Limited evidence suggests that helmet NIV may reduce the risk of transmission to HCW [2]. A retrospective study which examined transmission risk of SARS to HCW in relation to patient’s mode of ventilation found that among HCW who developed SARS, 38% had been exposed to patients receiving NIV, 35% to patients undergoing intubation and mechanical ventilation, and 8% to patients receiving HFNC [3]. However, in a separate study of patients with acute respiratory failure secondary to SARS receiving NIV under strict conditions of patient isolation, adequate airflow, full personal protective equipment (PPE) of HCW, and placement of a viral–bacterial filter between the mask and the exhalation port, there were no HCW infections [4]. However, the primary complication associated with filter use, as discussed previously, is interference with ventilator function.

One of the feared complications of NIV in patients with transmissible respiratory infections is air leakage from an imperfect seal resulting in aerosolization of infective pathogens and a resultant superspreading event [5–7]. This concern is particularly relevant in COVID-19 in which the virus can remain actively infectious in aerosols for up to 3 h and is more enduring on plastic with evidence of viable virus detectable up to 72 h following surface exposure [8]. A study examining the dispersion of exhaled air via different ventilatory support strategies found CPAP via oronasal mask and NIV via helmet resulted in negligible air dispersion, whereas nasal cannula oxygen at 5 L/min resulted in dispersion up to 1 m [9]. This study was completed in a negative pressure environment, thus results are not entirely generalizable. Another study of exhaled air dispersion found that the use of HFNC and CPAP with nasal pillows were both associated with exhaled air dispersion; however, air dispersion was negligible when CPAP was administered through an oronasal mask [10].

When utilizing HFNC, higher flows are likely to increase risk of aerosolization. The dispersion distance associated with HFNC use increased from 6.5 ± 1.5 cm at a flow of 10 L/min to 17.2 ± 3.3 cm at a flow of 60 L/min [10]. These are in comparison to a dispersion distance of 9.5 ± 0.6 cm for face mask at 10 L/min and a distance of 24.6 ± 2.2 cm for a nonrebreather mask at 10 L/min [11]. When using HFNC in pandemic medicine, it is advised to start at lower flows and as flow requirements increase it may be prudent to consider alternative respiratory strategies. It is also recommended that patients on HFNC with nasal prongs wear a surgical mask to limit exhaled air dispersion [12, 13]. Loh et al. evaluated the impact of HFNC on dispersion distance when coughing and reported that cough droplets spread to a mean distance of 2.48 m at baseline, 2.91 m with HFNC at 60 L/min, and a maximum recorded distance of 4.5 m [14]. Leung et al. studied environmental bacterial contamination in pneumonia patients and found that HFNC, when compared to a standard oxygen mask, did not result in increased air or surface contamination by gram-negative bacteria [15].

In pandemic medicine, the use of a dual limb circuit for CPAP and NIV is far superior to a single limb circuit as the dual limb circuit is a closed system. If a single limb circuit is to be utilized, a viral filter should be placed over the leak port to further reduce the risk of aerosol dispersion. The lowest possible pressures should be used with CPAP and NIV, and the lowest possible flows with HFNC. While limiting the risk of aerosol dispersion is of clinical importance, in a review of HFNC use in COVID-19, Lyons and Callaghan highlight the lack of current evidence regarding any clinically meaningful relationship between increased aerosolization and increased risk of spread to HCW [16]. On the contrary, recent evidence suggests that HFNC might not increase the risk of fugitive bioaerosols. Bem et al. quantified aerosol generation using laser light scattering and a particle counter and found that HFNC was not associated with increased aerosol generation compared to conventional oxygen therapy in both healthy individuals and in those with acute respiratory disease, including COVID-19 [17]. A recent editorial by Li and Scott reviewing current literature also suggests that the risk of transmission of COVID-19 with HFNC use is quite low [18]. Nevertheless, future research is necessary to more accurately quantify the degree of risk associated with HFNC use in COVID-19.

## Historical use and efficacy of noninvasive respiratory support

In Additional file 2: Text S2, we review the effectiveness of NIRS in several disease states such as acute hypoxemic respiratory failure, ARDS, pneumonia, and past viral epidemics. We also compare the efficacy of different delivery methods of NIV.

## Noninvasive respiratory support in COVID-19

It is well known that the clinical presentation of COVID-19 varies drastically, from asymptomatic to severe ARDS, multiorgan system failure and death. While it has been hypothesized that there may be different COVID-19 phenotypes that could explain the variance in clinical presentation, more evidence is needed to support those claims. Nevertheless, the heterogeneity witnessed in disease severity and presentation can make initial treatment challenging. It is known from studies in mechanically ventilated patients with ARDS that ventilation with high tidal volumes and elevated driving pressures may induce ventilator induced lung injury. In spontaneously breathing patients we may expect similar consequences if patients are breathing with large driving pressures and large tidal volumes without being appropriately monitored. Based on this concept, the traditional term ventilator-induced lung injury has been modified by some authors into *ventilation-induced* lung injury, to underline the fact that it is not the ventilator itself injuring the lung, but rather the unprotective ventilation. Along the same line, the concept of patient-self-inflicted lung injury (P-SILI) has been developed to describe the potential injurious ventilation in spontaneously breathing patients.

A recent study showed that the beneficial effect of lowering the tidal volume on mortality in mechanically ventilated patients with ARDS varies according to elastance, suggesting that lung protective ventilation strategies should target driving pressure rather than tidal volumes [19]. Whether this translates to spontaneously breathing patients is still unknown; however, this observation might be particularly relevant for patients with early COVID-19 ARDS, in which respiratory system compliance is often higher than in “classic” ARDS. A recent study showed that spontaneously breathing patients with COVID-19 ARDS have lower values of inspiratory effort as assessed by delta esophageal pressure, lower respiratory rate and lower minute volume ventilation as compared with patients with a similar degree of hypoxemia due to “classic” ARDS [20].

Ideally, to minimize the risk of injurious ventilation during spontaneous breathing, both tidal volume and driving pressure should be monitored. The use of esophageal manometry can help in determining the patient’s work of breathing and estimate the driving pressure, although it requires placement of an esophageal balloon catheter in awake patients with respiratory distress and it is not yet considered a standard of care. While HFNC does not allow monitoring of tidal volume, NIV would provide some tidal volume measurement, although leaks at the NIV interface might affect measurements. Patients who are breathing with tidal volumes larger than 9 mg/kg should be considered high risk for NIRS failure, and IMV should be strongly considered [21]. However, early intubation and mechanical ventilation for all patients at risk for P-SILI may not be the solution either, as there are several risks associated with IMV, including ventilator associated pneumonia, ventilator induced lung injury, prolonged sedation, prolonged immobility, and muscle wasting. Further research is needed to deepen our understanding of the role of P-SILI in COVID-19, and whether or not early intubation is an effective treatment strategy to reduce the incidence of P-SILI.

There remains limited data pertaining to the efficacy and safety of NIRS in the management of COVID-19. A prospective cohort study out of New York found that 22% of patients admitted with COVID-19 were critically ill and 79% of those critically ill patients required IMV [22]. Of those patients who required IMV, 62% had first received some form of supplemental oxygen or NIRS (non-rebreather, HFNC or NIV) [22]. In comparison, a retrospective cohort study of COVID-19 patients in Wuhan, China found development of ARDS in 41.8% of admitted patients, ICU admission in 26.4%, and death in 21.9% [23]. Of note, China took a different approach to oxygen therapy in COVID-19 patients, preferring noninvasive support over IMV. Of all patients hospitalized in the study, 48.8% received nasal cannula, 30.3% received some form of NIRS, and a mere 2.5% received IMV [23]. Of those patients who developed ARDS and survived, 42.5% had received nasal cannula and 57.5% had received NIRS [23]. Of those patients who developed ARDS and died, 86.4% had received NIRS and only 11.4% had received IMV [23]. A retrospective study of 318 COVID-19 patients in Chongqing, China found that 41% of patients admitted with ARF managed with HFNC as first line therapy experienced HFNC failure and required NIV as rescue therapy [24]. Of clinical importance, the HFNC failure rate was 0% in patients with PaO_2_/FiO_2_ > 200 mmHg and 63% in those with PaO_2_/FiO_2_ ≤ 200 mmHg [24]. In addition, it was noted that the respiratory rate (RR) significantly decreased after 1–2 h of HFNC in the successful group but not in the unsuccessful group [24]. Of those who failed HFNC and required NIV as rescue therapy, PaO_2_/FiO_2_ significantly improved after 1–2 h of NIV; however, 29% of those patients ultimately required IMV [24]. This suggests that the rate of HFNC and NIV failure is not insignificant, which is especially appreciated in patients with severe ARDS.

Coppadoro et al. conducted an observational study to evaluate the efficacy of helmet CPAP on COVID-19 patients (study included both full code and DNI patients) who had failed standard oxygen therapy. Helmet CPAP was successful in treating 69% of full code patients and 28% of DNI patients [25]. Helmet CPAP was associated with significant improvement in oxygenation (PaO_2_/FiO_2_ increase of approximately 100) and respiratory distress (RR decrease from 28 to 24) [25]. Ing et al. suggest that helmet CPAP may be a safe, effective strategy in the management of hypoxemic respiratory failure secondary to COVID-19 so long as the patient is not showing signs of excessive inspiratory work or development of classic ARDS [26]. The HENIVOT trial by Grieco et al. evaluated the use of helmet NIV followed by HFNC versus HFNC alone in the management of COVID-19 respiratory failure and found that there was no significant difference in median days free of respiratory support between groups; however, the helmet NIV group had significantly lower rates of IMV [27]. Duan et al. conducted a multicenter retrospective study of COVID-19 patients who received either HFNC or NIV as first line therapy and found no significant difference between groups in duration of NIRS, intubation, or mortality [28].

More recent literature pertaining to acute respiratory failure secondary to COVID-19 has the added benefit of providing clinicians with evidence-based outcomes that can help guide clinical decision-making. Liu et al. conducted a retrospective, multicenter observational study in an effort to develop a nomogram that would predict likelihood of NIRS failure in COVID-19 patients and found that predictors of NIRS failure included age, number of comorbidities, GCS score, ROX index, and vasopressor use on day one of NIRS [29]. Menga et al. conducted a prospective observational study evaluating the rate of NIRS failure in COVID-19 patients and reported a failure rate of 61%, with failure defined as the need for IMV [30]. Interestingly, patients with hypoxemic respiratory failure secondary to COVID-19 were nearly twice as likely to fail a trial of NIRS compared to patients with non-COVID related hypoxemic respiratory failure [30]. Improvement in the PaO_2_/FiO_2_ ratio after one hour of NIRS was not predictive of NIRS success, whereas independent predictors of NIRS failure included SAPS II greater than 33 and LDH greater than 405 [30]. Hill and Devaraj hypothesize that the difference in NIRS failure rates between COVID and non-COVID-related hypoxemic respiratory failure may be due to the cytokine storm and subsequent multi-organ system failure associated with COVID-19 [31]. This may also explain why the initial improvement frequently seen in﻿ PaO_2_/FiO_2_ ratio in COVID-19 patients on a trial of NIRS is not consistently predictive of NIRS success [31]. The unique clinical course associated with COVID-19 hypoxemic respiratory failure is evidence that COVID-19 cannot be treated in the same manner as previous forms of hypoxemic respiratory failure, and that careful monitoring of patients on a trial of NIRS is required to prevent adverse outcomes associated with delayed intubation [31].

### Prone positioning

One of the strategies utilized in the fight against COVID-19 that has potential widespread implications for future management is awake prone positioning, both with and without concurrent use of NIRS, as reviewed in Additional file 3: Text S3.

### Professional society guidelines for the use of NIRS in COVID-19

The World Health Organization conducted a systematic review of ventilation strategies for coronavirus (the review included MERS, SARS and COVID-19) and concluded that NIV may reduce mortality and need for intubation, but that it also has the potential to increase spread to HCW [2]. Societal guidelines for the use of NIV as first line therapy for the management of COVID-19 have been evolving as our understanding of the disease grows. HFNC is considered first line therapy by the Spanish Society of Pneumology and Thoracic Surgery, the European Society of Intensive Medicine, the Society of Critical Care Medicine, the Chinese Thoracic Society, and the Australian and New Zealand Intensive Care Society [32]. CPAP is considered first line therapy by the Italian Association of Hospital Pulmonologists (specifically helmet CPAP) and the National Health Service. HFNC or CPAP is recommended by the Portuguese Society of Pulmonology, HFNC or NIV by the World Health Organization, and helmet NIV by multiple German societies [32]. Recently updated SCCM guidelines provide a weak recommendation in favor of HFNC over NIV in patients with respiratory failure despite conventional oxygen [33]. SCCM also provides a weak recommendation in favor of NIV if HFNC is not available and there is no urgent indication for intubation [33].

## Implementation of noninvasive respiratory support in COVID-19

Based on the literature review provided above, we posit that NIRS, when utilized in the appropriate setting, is an appropriate alternative to early IMV for patients presenting to the hospital with suspected or confirmed COVID-19. However, stringent observation is necessary with NIRS to allow for early detection of clinical deterioration as NIRS failure is associated with increased risk of hospital mortality, ICU stay and hospital stay [34]. A recent publication by Raoof et al. outlines several indications and techniques for the use of NIRS in COVID-19 patients based on clinical presentation and symptom severity [35]. Raoof et al. advocate for supplemental oxygen in patients with no respiratory distress but with SpO_2_ < 92–94% on room air (RA) or declining SpO_2_, with escalation to NIRS in patients with mild to moderate respiratory distress, increased work of breathing, PaO_2_/FiO_2_ > 150 but < 300, or SpO_2_ < 90–94% on non-rebreather [35]. They propose immediate IMV in patients with severe respiratory distress, PaO_2_/FiO_2_ < 150, or SpO_2_/FiO_2_ < 196 [35]. There is a large body of evidence in the literature pertaining to factors that predict NIV failure. These can be broadly divided into four categories. First, patient specific risk factors for NIRS failure include high APACHE II, high SAPS II, high SOFA, older age, multiorgan dysfunction, mask intolerance, poorly controlled respiratory secretions, neurologic impairment (measured via GCS), ARDS, pneumonia, worsening chest imaging, and failure to improve clinically after 1 h of NIRS [34, 36–42]. Second, laboratory values predictive of NIV failure include pH < 7.25 or PaCO_2_ > 75 (in hypercapnic ARF) after two hours of NIV, lack of improvement in blood gas, lower bicarbonate, lower PaCO_2_ (in hypoxemic ARF), higher lactate, and failure to maintain a PaO_2_ of 60 mmHg on FiO_2_ of 0.6 [26, 34, 36–42]. Third, ventilatory predictors of NIV failure include PaO_2_/FiO_2_ < 150–200, a tidal volume of > 9.0–9.5 ml/kg of predicted body weight, and high peak pressure requirement [21, 26, 39–41, 43]. Finally, vital sign trends that are concerning for impending NIRS failure include increasing tachycardia, hemodynamic deterioration, and worsening dyspnea/tachypnea [26, 38–41].

- Table 1 provides a list of indications for HFNC and NIV/CPAP in patients presenting to the hospital with COVID-19. NIV may be particularly beneficial for individuals with comorbid conditions such as COPD, obstructive sleep apnea, hypercapneic respiratory failure, cardiogenic pulmonary edema, CHF, and OSA. Table 2 provides our list of contraindications to NIRS. Due to the speed with which COVID-19 patients can deteriorate, immediate intubation is warranted for any individual who presents with altered mental status, hemodynamic instability, multiorgan dysfunction, or severe hypoxaemia defined by an SpO_2_ of < 80%, while on supplemental oxygen. Table 3 outlines our approach to the timely monitoring of COVID-19 patients undergoing a trial of NIRS. To prevent rapid and unpredicted patient decompensation, we recommend close monitoring for the first 3 h. If a patient tolerates NIRS for 3 h without evidence of clinical deterioration, frequency of assessments can be liberalized based on the patient's condition and the physician’s clinical judgement. Table 4 outlines multiple indicators of pending NIRS failure. As the number of indicators from Table 4 increases in a particular patient over time, the higher the likelihood of NIRS failure. Ultimately, however, these tables are meant to serve primarily as an adjunct and cognitive aid in the decision-making process. Table 5 outlines institutional level considerations to maximize the safety profile of NIRS and to limit spread to HCW. To minimize risk to health care workers, any COVID-19 patient receiving supplemental oxygen should be placed in an airborne isolation room if possible and staff caring for the patient should use full contact, droplet, and airborne isolation precautions [44, 45].

**Table 1:** Indications for NIV and HFNC in the setting of Acute Respiratory Failure

Indications for NIV in the Setting of Acute Respiratory Failure 1) Known patient history of OSA, COPD, congestive heart failure, or cardiogenic pulmonary edema [46, 47] 2) Hypercapnic respiratory failure 3) Dyspnea or staccato speech [48, 49]
Indications for HFNC in the Setting of Acute Respiratory Failure 1) PaO_2_ < 65 or﻿ SpO_2_ < 90% on supplemental oxygen [48] 2) RR > 25 [49] 3) Mild ARDS as defined by PaO_2_/FiO_2_ < 300 but > 200 [24, 49]

**Table 2:** Contraindication to Non-invasive Ventilation (NIV)

Contraindications to NIV
1) Cardiac and respiratory arrest 2) Encephalopathy or altered mentation [37] 3) Severe hypoxaemia on admission defined as﻿ PaO_2_/FiO_2_ < 150 [50] 4) Pneumothorax, pleural effusion, or pulmonary embolism [49] 5) Active upper gastrointestinal bleed, emesis, or aspiration risk [37] 6) Recent facial trauma or facial surgery [37] 7) Hemodynamic instability as defined by vasopressor use [37, 51] 8) Multiorgan dysfunction or failure [51] 9) SOFA score > 5 is predictive of NIV failure [51, 52] 10) Poorly controlled respiratory secretions [37, 39, 53] 11) CXR/CT showing evidence of bilateral, multilobar involvement [39, 51–53]

**Table 3:** Appropriate monitoring of Noninvasive Respiratory Support (NIRS)

Appropriate Monitoring of Noninvasive Respiratory Support
1) Hourly lab assessment (for 3 h) a) ABG including PaO_2_, PaCO_2_, bicarbonate, lactate, and base excess b﻿) PaO_2_/FiO_2_ (target PaO_2_/FiO_2_ > 300) [24, 50] c) Subjective improvement or worsening of dyspnea [4] 2) Continuous monitoring (for 3 h): a) Heart rate and respiratory rate trends [4, 24] b) Pulse oximetry and FiO_2_ requirement c) Tidal volume measurement if utilizing CPAP or NIV [21, 43, 54]

**Table 4:** Primary and Secondary Indicators of Noninvasive Respiratory (NIRS) failure

Primary Indicators of Noninvasive Respiratory Support Failure 1)﻿ PaO_2_/FiO_2_ < 150 or inability to improve PaO_2_/FiO_2_ after 1 h of NIV [39, 50, 55] 2) Worsening/unimproved dyspnea or tachypnea > 25 after 1 h of NIV [24, 39, 53, 56] 3) Failure to maintain PaO_2_ of 60 on FiO_2_ of 0.6 [39, 53] 4)﻿ SpO_2_/FiO_2_ < 196 [35] 5) Tidal volume of > 9 ml/kg predicted body weight [21, 43, 54] 6) ROX value less than 2.85 at 2 h, less than 3.47 at 6 h, or less than 3.85 at 12 h predict HFNC failure [57] 7) pH < 7.25 or PaCO_2_ > 75 after 2 h of NIV [42]
Secondary Indicators of Noninvasive Respiratory Support Failure 1) SAPS II > 35, APACHE II > 17, or rising SOFA score [39, 51, 52, 55] 2) High peak pressure requirement [39, 53] 3) Worsening bronchorrhea [39, 53] 4) Intolerance of mask [39, 53]

**Table 5:** Safety considerations for Noninvasive Respiratory Support (NIRS) in COVID patients

Safety Considerations for Noninvasive Respiratory Support in COVID patients
1) Isolated negative pressure environment (room, hood, tent) [44] a) Preferably with anteroom and private bathroom 2) Full contact, droplet, and airborne isolation precautions [44] 3) Full PPE that includes PAPR or N-95, gown, gloves, and face/eye shield [4] 4) Escalation of care to ICU for rapidly increasing O_2_ requirement or patients on NIV 5) NIV with helmet and tight air cushion or unvented oronasal mask [9] a) Dual limb circuit over single limb circuits when utilizing CPAP or NIV 6) For single limb circuit, filter over leak port 7) Viral–bacterial filter between mask and exhalation port [4] 8) Staffing that allows for close monitoring to assess for deterioration 9) Sterile equipment nearby in preparation for emergent intubation in the event of rapid deterioration 10) Daily monitoring of HCW for symptoms[1]

**Fig. 1:**
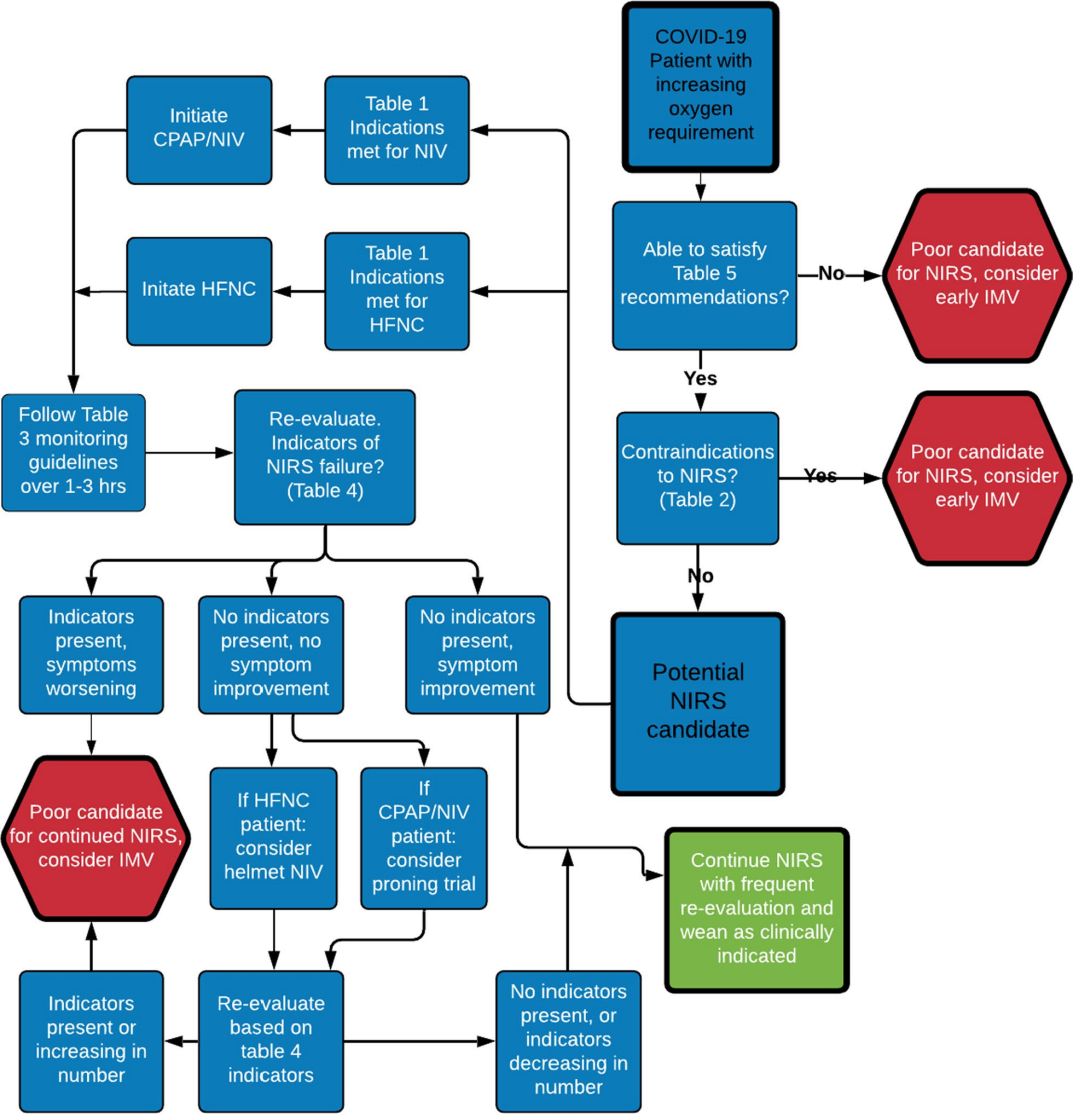
Proposed Noninvasive Respiratory Support (NIRS) Algorithm

In an effort to develop a cognitive aid in the clinical decision-making process, Tables 1, 2, 3, 4 and 5 were used to create an algorithm (Fig. 1) for clinicians considering the use of NIRS. The primary aim of the algorithm is to assist clinicians in answering three clinical questions: 1) Is the patient a candidate for NIRS? 2) If the patient is a candidate, which NIRS modality should be utilized? and 3) What factors should be used to identify NIRS failure requiring IMV? In order for a patient to be considered a candidate for NIRS, the criteria in Table 4 should be met and there should be no contraindications present from Table 2. Per Society of Critical Care Medicine (SCCM) guidelines, initiation of HFNC over NIV is appropriate unless one of the specific indications for NIV in Table 1 is met, in which case NIV may be utilized over HFNC. Once NIRS is initiated, close monitoring should be conducted for approximately 3 h, with a focus on HR, RR, hemodynamic stability, tidal volumes, and repeat blood gases to assess PaO_2_, PaCO_2_, and PaO_2_/FiO_2_ ratio. If at any point the patient begins to decompensate, develops a contraindication to NIRS (Table 2), or shows evidence of NIRS failure (Table 4), NIRS should be aborted in favor of immediate IMV. After the initial 3 h NIRS trial, if the patient has improved, it is appropriate to continue NIRS and wean as clinically indicated. If, however, the patient remains stable without evidence of significant improvement, the clinician may consider a trial of NIRS with prone positioning, with the understanding that there remains no recommendation in favor of or against prone positioning for COVID-19 from the SCCM due to a current lack of demonstrable evidence in its favor [33]. If the clinician determines the patient is a good candidate for a proning trial with NIRS, close monitoring for deterioration should be continued. Deterioration or lack of symptomatic improvement (as outlined by Tables 2, 3 and 4) after a proning trial should trigger escalation of care to IMV. See image one below for the associated algorithm. The algorithm is not meant to outline precisely which patients should and should not receive NIRS as there is a current lack of clinical evidence to that end; rather, the goal of the algorithm is to provide a working framework of evidence based cautions, contraindications, and management techniques for those physicians considering the use of NIRS in COVID-19 patients.

## Future considerations

There appears to be a role for noninvasive respiratory support in the context of acute hypoxemic respiratory failure secondary to COVID-19, although more research is indicated to deepen our understanding of the precise benefits and risks associated with NIV for not only patients but also HCW [32]. A prospective RCT assessing outcomes such as intubation rate, ICU length of stay, and mortality in COVID-19 patients who are randomly assigned to one of two groups (immediate IMV vs. NIRS) with further subgroup analysis of outcomes for patients who fail NIRS and require IMV would go a long way in not only parsing out the efficacy of NIRS in COVID-19 patients, but would also provide insight into whether patients intubated following NIRS failure have better, worse, or equivocal outcomes compared to patients immediately intubated. As the COVID-19 pandemic continues to evolve, a stringent focus on which interventions objectively improve outcomes will be a valuable tool for clinicians, especially in situations of limited resources. Until that time, the use of NIRS in the management of COVID-19 can be considered safe and appropriate, particularly in the setting of potential ventilator shortages, when it is administered under the supervision of clinicians who understand not only the associated benefits and risks, but also when to appropriately transition to IMV.

## Conclusion

Until future research provides clinically significant evidence pertaining to the efficacy of NIRS in the management of COVID-19, the judicious use of NIRS in select patients should be considered. A systems-based approach to the use of NIRS is strongly recommended and the safety practices outlined above would provide HCW with a significant degree of protection as we collectively work towards minimizing the likelihood of healthcare associated COVID-19 dissemination. When utilized with vigilance and under appropriate conditions, NIRS is an acceptable alternative to early IMV in the management of mild to moderate acute hypoxemic respiratory failure secondary to COVID-19**.**

## Take home message

The mortality rate and prolonged ventilator days associated with invasive mechanical ventilation (IMV) of patients with severe COVID-19 have incited a debate surrounding the use of noninvasive respiratory support (NIRS) (i.e., HFNC, CPAP, NIV) as a potential treatment strategy. We review the existing literature with a focus on rationale, patient selection and outcomes associated with the use of NIRS in COVID-19 and prior pandemics, as well as in patients with acute respiratory failure due to different etiologies (i.e., COPD, cardiogenic pulmonary edema, etc.) to understand the potential role of NIRS in COVID-19 patients. Based on this analysis we suggest an algorithm for NIRS in COVID-19 patients which includes indications and contraindications for use, monitoring recommendations, systems-based practices to reduce healthcare worker (HCW) exposure, and predictors of NIRS failure.

## Supplementary Information

**Additional file 1: Text S1.** Noninvasive respiratory support strategies.

**Additional file 2: Text S2.** Historical use and efficacy of noninvasive respiratory support.

**Additional file 3: Text S3.** Prone positioning.

## Data Availability

Not applicable.
