# Supplementary material for: Noninvasive respiratory support for COVID-19 patients: when, for whom, and how?
Source: J Intensive Care. 2022 Jan 15;10:3. doi: 10.1186/s40560-021-00593-1 (PMC8760575; doi:10.1186/s40560-021-00593-1)
Supplement: Supplementary file 1 — Additional file 1: Text S1. Noninvasive respiratory support strategies. [file 40560_2021_593_MOESM1_ESM.docx]

**Noninvasive Respiratory Support Strategies**

There are several NIRS strategies available to clinicians and understanding the differences between each is crucial when determining the optimal treatment method for patients presenting with acute respiratory failure (ARF) secondary to COVID-19. Thus, it is important to briefly outline the most clinically relevant types of NIRS, the risks and benefits of each, and the different delivery methods available. Broadly speaking, the most commonly used NIRS strategies are high flow nasal cannula (HFNC), continuous positive airway pressure (CPAP), and non-invasive ventilation (NIV).

*High Flow Nasal Cannula (HFNC)*

HFNC is administered via two nasal prongs that are secured to the patient’s head in a manner that does not provide a tight fitting seal around the patient’s face and may increase the risk of aerosolization compared to the nasal/oronasal mask and helmets used for CPAP and NIV (1). However, HFNC was found to be more comfortable and require less medical staffing when compared to NIV (2). When administering HFNC, the two titratable parameters are flow and inspired fraction of oxygen (FiO_2_). Most commonly, the maximum flow rate is 60 L/min and the maximum FiO_2_ is 1. By providing high flow humidified oxygen, HFNC results in advantageous physiological effects including reduced upper airway dead space (3) and a flow dependent positive end-expiratory pressure (PEEP) of up to 7 cm H2O at 50 L/min of flow and mouth closed (4). These effects have been shown to result in reduced respiratory rate (1, 5), improved oxygenation (1, 5), decreased dyspnea (1, 6), and decreased mouth dryness (1). While the administration of HFNC and humidified oxygen affords patients greater comfort and improved airway clearance, it does not allow easy measurement of tidal volume and minute ventilation. Therefore, there is a risk of high tidal volumes and transpulmonary pressures, which might increase the risk of self-inflicted lung injury.

For patients receiving HFNC, the ROX index has been validated as a tool to assess the likelihood of HFNC failure in patients with acute hypoxemic respiratory failure. The ROX index is calculated by first dividing oxygen saturation by FiO_2_; the quotient is then divided by the respiratory rate. A study evaluating patients with acute respiratory failure secondary to pneumonia was conducted to determine if the ROX index could reliably assess the likelihood a patient would fail HFNC and require intubation (7). Investigators found that a ROX value greater than 4.88 calculated at 2, 6, or 12 hours following initiation of HFNC was associated with a significantly lower risk of intubation (7). A ROX value above 4.88, regardless of time since onset of HFNC, appears to be the most important marker associated with HFNC success (7, 8). The positive predictive value (PPV) of a ROX value above 4.88 at 2 and 6 hours was over 75%, and the PPV of a ROX above 4.88 at 12 hours and beyond was over 80% (7). A ROX value less than 2.85 at 2 hours (99.2% specificity), less than 3.47 at 6 hours (99.2% specificity), or less than 3.85 at 12 hours (98.4% specificity) were all predictors of HFNC failure (7). Compared to patients with a successful trial of HFNC, those who failed HFNC tended to have smaller increases in their ROX values from 2-12 hours and again from 12-24 hours (7). Interestingly, patients who required intubation after 12 hours or more of HFNC had an increased relative risk of hospital mortality compared to patients intubated <12 hours after initiating HFNC (7).

The ROX index has also been found to be an independent predictor of HFNC failure in COVID-19 patients (9, 10). A retrospective review by Chandel et al assessed the utility of using the ROX index to predict HFNC success in COVID-19 patients and found that a ROX index greater than 3 was 85.3% sensitive for predicting HFNC success (9). Mortality rate was high in patients who failed HFNC (45.3%), with a hospital mortality of 39.3% in early HFNC failure (as defined by failure less than 48 hours after initiation) vs. 53.2% in late HFNC failure, although this finding was not deemed statistically significant (9). This data suggests that the ROX index is a highly efficacious tool for clinicians who want to trial HFNC while simultaneously assessing the likelihood of HFNC failure within as early as two hours of initiation.

HFNC utilization may result in delayed intubation for patients, which is not without its own risks. A study evaluating outcomes associated with HFNC failure split HFNC patients into two groups, the early intubation group (<48 hours after HFNC initiation) and delayed intubation group (> 48 hours after onset of HFNC) (11). The delayed intubation group had higher ICU mortality, more days of ventilation, and fewer ventilator free days (11). However, a systematic review and meta-analysis comparing HFNC to conventional oxygen therapy in patients with acute hypoxemic respiratory failure found decreased rates of intubation and decreased escalation of oxygen therapy in the HFNC group; no significant differences were noted in mortality, ICU length of stay, and patient reported comfort or dyspnea (12). This evidence suggests that HFNC may decrease intubation rates in hypoxemic respiratory failure, but that delayed intubation may increase ICU mortality. An added benefit of HFNC administration is that it has the potential to free up ventilators in regions struggling with ventilator shortages. Gershengorn et al developed dynamical simulation models in an effort to optimize HFNC and IMV use in COVID-19 patients and found that a two-pronged approach which combined HFNC use in patients not urgently requiring IMV with early intubation in patients when at least 10% of ventilator supply was not in use resulted in fewer deaths and increased ventilator availability (13).

*Continuous Positive Airway Pressure (CPAP) and Noninvasive Ventilation (NIV)*

CPAP and NIV are most frequently delivered via an oronasal mask that is intended to maintain a seal around the patient’s nose and mouth with the risk of aerosolization dependent on any leaks in the seal. Vented masks may also be used; however, they increase the risk of fugitive aerosols. The nasal mask, an alternative to the oronasal mask for CPAP use,

creates a tight seal around the patient’s nose while leaving the mouth uncovered. CPAP and NIV can also be delivered via a helmet or face mask which creates an artificial environment surrounding the patient’s head and neck to maximize pressure support and minimize aerosolization risk. CPAP delivers a preset pressure that is constant during both inhalation and exhalation. NIV, in addition to providing PEEP during exhalation, also administers a predetermined pressure (or volume) during inspiration. NIV can be delivered via multiple modes (pressure support, volume control, bilevel positive airway pressure) but is most commonly administered as bilevel positive airway pressure.

The circuit used for CPAP and NIV impacts the risk of aerosolization. Patients are connected to a ventilator through either a single limb or dual limb circuit. The dual limb circuit is a closed system that has one inhalational limb and one exhalation limb, similar to the type of circuitry used in IMV. The single limb circuit has one inhalational limb with either a passive exhalation port or an active exhalation valve, both of which are located on the distal aspect of the circuit. A heat and moisture exchange filter can be placed between the circuit and the patient; however, the use of filters can interfere with ventilator function. The passive leak port increases the risk of fugitive aerosols, unless covered by a filter, and it can be placed in either the interface or the circuit. In contrast, the active exhalation valve is a closed circuit and allows for placement of a filter on the valve. Utilization of a single limb circuit is discouraged as it increases risk of aerosolization when compared to dual limb circuits. Patout et al assessed the effect of various circuit setups on ventilator function during NIRS and found the optimal setup to be a dual-limb circuit with an oronasal mask and the worst setup to be a dual-limb circuit with a helmet interface (14). The circuit setup, along with the use of a filter, had a significant impact on multiple variables including inspiratory effort needed to trigger the ventilator, inspiratory flow prior to trigger, maximal inspiratory pressure, tidal volume, and work of breathing (14). While the risk of aerosolization for CPAP and NIV (with the use of a dual limb circuit and good fit) may be less than HFNC, CPAP and NIV are less comfortable and less tolerated (1). Furthermore, the staffing and time requirements necessary for the safe and judicious use of NIV are not trivial. Efforts to utilize CPAP or NIV are largely dependent on each hospital’s ability to adequately staff wards where these treatment methods are being delivered, a challenge that is compounded in pandemic medicine when staffing is overwhelmed and resources are limited.

In 2017 the American Thoracic Society and the European Respiratory Society published comprehensive guidelines for the use of NIV in the management of acute respiratory failure due to numerous etiologies. A strong recommendation in favor of NIV was made for cardiogenic pulmonary edema and hypercapnic respiratory failure in COPD (15). A conditional recommendation for NIV was made in the setting of an immunocompromised state, trauma, post-operatively, and in palliative care (15). No recommendation was made for the utilization of NIV in acute asthma exacerbation, de novo ARF, and importantly, in the management of ARF due to a pandemic viral illness (15).

**References**

1. Roca O, Riera J, Torres F, Masclans JR. High-flow oxygen therapy in acute respiratory failure. Respir Care. 2010;55(4):408-13.

2. Xu Q, Wang T, Qin X, Jie Y, Zha L, Lu W. Early awake prone position combined with high-flow nasal oxygen therapy in severe COVID-19: a case series. Crit Care. 2020;24(1):250.

3. Moller W, Celik G, Feng S, Bartenstein P, Meyer G, Oliver E, et al. Nasal high flow clears anatomical dead space in upper airway models. J Appl Physiol (1985). 2015;118(12):1525-32.

4. Ritchie JE, Williams AB, Gerard C, Hockey H. Evaluation of a humidified nasal high-flow oxygen system, using oxygraphy, capnography and measurement of upper airway pressures. Anaesth Intensive Care. 2011;39(6):1103-10.

5. Corley A, Caruana LR, Barnett AG, Tronstad O, Fraser JF. Oxygen delivery through high-flow nasal cannulae increase end-expiratory lung volume and reduce respiratory rate in post-cardiac surgical patients. Br J Anaesth. 2011;107(6):998-1004.

6. Rittayamai N, Tscheikuna J, Praphruetkit N, Kijpinyochai S. Use of High-Flow Nasal Cannula for Acute Dyspnea and Hypoxemia in the Emergency Department. Respir Care. 2015;60(10):1377-82.

7. Roca O, Caralt B, Messika J, Samper M, Sztrymf B, Hernandez G, et al. An Index Combining Respiratory Rate and Oxygenation to Predict Outcome of Nasal High-Flow Therapy. Am J Respir Crit Care Med. 2019;199(11):1368-76.

8. Roca O, Messika J, Caralt B, Garcia-de-Acilu M, Sztrymf B, Ricard JD, et al. Predicting success of high-flow nasal cannula in pneumonia patients with hypoxemic respiratory failure: The utility of the ROX index. J Crit Care. 2016;35:200-5.

9. Chandel A, Patolia S, Brown AW, Collins AC, Sahjwani D, Khangoora V, et al. High-Flow Nasal Cannula Therapy in COVID-19: Using the ROX Index to Predict Success. Respir Care. 2021;66(6):909-19.

10. Liu L, Xie J, Wu W, Chen H, Li S, He H, et al. A simple nomogram for predicting failure of non-invasive respiratory strategies in adults with COVID-19: a retrospective multicentre study. Lancet Digit Health. 2021;3(3):e166-e74.

11. Kang BJ, Koh Y, Lim CM, Huh JW, Baek S, Han M, et al. Failure of high-flow nasal cannula therapy may delay intubation and increase mortality. Intensive Care Med. 2015;41(4):623-32.

12. Rochwerg B, Granton D, Wang DX, Helviz Y, Einav S, Frat JP, et al. High flow nasal cannula compared with conventional oxygen therapy for acute hypoxemic respiratory failure: a systematic review and meta-analysis. Intensive Care Med. 2019;45(5):563-72.

13. Gershengorn HB, Hu Y, Chen JT, Hsieh SJ, Dong J, Gong MN, et al. The Impact of High-Flow Nasal Cannula Use on Patient Mortality and the Availability of Mechanical Ventilators in COVID-19. Ann Am Thorac Soc. 2021;18(4):623-31.

14. Patout M, Fresnel E, Lujan M, Rabec C, Carlucci A, Razakamanantsoa L, et al. Recommended Approaches to Minimize Aerosol Dispersion of SARS-CoV-2 During Noninvasive Ventilatory Support Can Cause Ventilator Performance Deterioration: A Benchmark Comparative Study. Chest. 2021;160(1):175-86.

15. Rochwerg B, Brochard L, Elliott MW, Hess D, Hill NS, Nava S, et al. Official ERS/ATS clinical practice guidelines: noninvasive ventilation for acute respiratory failure. Eur Respir J. 2017;50(2).
