# Supplementary material for: Noninvasive respiratory support for COVID-19 patients: when, for whom, and how?
Source: J Intensive Care. 2022 Jan 15;10:3. doi: 10.1186/s40560-021-00593-1 (PMC8760575; doi:10.1186/s40560-021-00593-1)
Supplement: Supplementary file 2 — Additional file 2: Text S2. Historical use and efficacy of noninvasive respiratory support. [file 40560_2021_593_MOESM2_ESM.docx]

**Historical Use and Efficacy of Noninvasive Respiratory Support**

While the scientific community works toward a more comprehensive understanding of the pathophysiology behind COVID-19, it is pertinent to review the effectiveness of NIRS in disease states such as pneumonia, ARDS, and past viral epidemics.

*Pneumonia*

Findings of studies examining the efficacy of NIV in the management of community acquired pneumonia (CAP) have been conflicting. Confalonieri et al studied NIV in patients with ARF (defined as PaO_2_/FiO_2_ ratio <250) secondary to CAP, including patients with and without COPD. When compared to standard treatment, NIV resulted in a significant decrease in respiratory rate, ICU stay, and rate of IMV; a subgroup analysis, however, showed that the benefits of NIV occurred only in those patients with COPD (1). In a review of NIV use in patients with HIV/AIDs and ARF due to pneumonia receiving NIV support, the following factors were identified as predictive of NIV failure: age > 40, ARDS, SAPS II >35, APACHE II >17, RR > 25 one hour after NIV, hemodynamic instability or shock, severe hypoxemia at admission, and PaO_2_/FiO_2_ <175 one hour after NIV initiation (2). In a meta-analysis, NIV appeared to have a benefit in patients with CAP and acute pulmonary edema (3). A separate meta-analysis comparing NIV to standard medical care in patients with community-acquired pneumonia found NIV resulted in a significantly reduced rate of intubation and ICU mortality, but had no effect on hospital mortality (4). These findings suggest that NIV may have a role in the management of CAP, especially in patients with comorbid COPD, OSA or cardiogenic pulmonary edema.

*Acute Hypoxemic Respiratory Failure and ARDS*

There remains mixed evidence for the utilization of NIRS in managing acute hypoxemic respiratory failure and ARDS. A substudy of the LUNG SAFE trial found NIV failure occurred in 22.2% of mild, 42.3% of moderate, and 47.1% of patients with severe ARDS (5). Hospital mortality was 16.1% in patients with NIV success and 45.4% in NIV failure (5). The use of NIV, regardless of success or failure, was correlated with increased ICU mortality (5). Importantly, a propensity matched analysis found that ICU mortality was higher in NIV than with IMV when PaO_2_/FiO_2_ <150 mm Hg (5). A prospective, multiple center cohort study found that NIV in ARDS avoided intubation in 54% of patients, with a SAPS II >34 and no improvement of PaO_2_/FiO_2_ after one hour as predictors of NIV failure (6). Tonelli et al assessed the relationship between inspiratory effort (measured by changes in esophageal pressure) and NIV failure in patients with hypoxemic respiratory failure and found that the tidal change in esophageal pressure after two hours of NIV was significantly lower in patients who completed a 24-hour trial of NIV compared to those who failed the trial and required IMV (7). They also found that a reduction in the tidal change in esophageal pressure of 10 cm H2O or more after two hours of NIV was associated with avoidance of intubation and success of NIV at 24 hours (7). A meta-analysis studying NIV in ARF found that NIV was associated with a significant decrease in mortality when used to prevent or treat ARF; however the mortality benefit was not present if NIV was used as rescue therapy rather than early in the disease course (8). In contrast, a separate meta-analysis by Agarwal et al found the addition of NIV to standard care in ARDS patients did not reduce the rate of IMV and had no effect on ICU mortality (9).

An alternative to NIV is HFNC, which has been found to reduce respiratory rate, reduce dyspnea, and improve oxygenation in patients presenting to the hospital with acute hypoxemic respiratory failure (10). The FLORALI trial from 2015 included 310 patients diagnosed with nonhypercapneic acute hypoxemic respiratory failure (secondary to pneumonia in most study patients) to evaluate differences in outcomes associated with the utilization of standard oxygen therapy, HFNC, and NIV (11). The authors found no significant difference in intubation rate between groups, however, the HFNC group had significantly more ventilator free days and a significantly lower 90 day mortality (11). Grieco et al conducted a randomized crossover study evaluating HFNC vs helmet NIV in the management of hypoxemic respiratory failure as defined by a PaO_2_/FiO_2_ ratio <200 and found that, compared to HFNC, helmet NIV improved PaO_2_/FiO_2_ and reduced respiratory effort, respiratory rate, and dyspnea (12). No significant differences were noted with transpulmonary pressure swings, PaCO_2_, and comfort (12). However, high transpulmonary pressure swings during inspiration with helmet NIV was associated with increased intubation rate and mortality (12). A recently published meta-analysis by Ferreyro et al evaluated the different rates of intubation and all-cause mortality in patients with hypoxemic respiratory failure based upon whether they received standard oxygen therapy, HFNC, helmet NIV, or face mask NIV (13). Helmet and face mask NIV, but not HFNC, were associated with significantly decreased all-cause mortality compared to standard oxygen therapy (13). In particular, helmet NIV was associated with decreased mortality compared to HFNC and face mask NIV (13). All three modalities studied were associated with lower risk of intubation compared to standard oxygen therapy (13). Similar to the mortality findings, helmet NIV was superior to both HFNC and face mask NIV in preventing intubation (13). In an effort to compare different NIV modes of delivery, Patel et al conducted a RCT to evaluate the use of face mask vs helmet NIV in ARDS patients and found a significant reduction in the intubation rate and 90 day mortality in the helmet NIV group (14). A recent systematic review and meta-analysis by Chaudhuri et al comparing helmet NIV to both facemask NIV and HFNC in acute respiratory failure found that helmet NIV may reduce mortality and intubation compared to facemask NIV for both hypoxic and hypercapnic respiratory failure (15). However, compared to HFNC, helmet NIV was not found to be superior (15). A systematic review and meta-analysis by Liu et al found that, compared to mask NIV, helmet NIV decreased hospital mortality and intubation rate with no significant difference in gas exchange in the management of acute respiratory failure (16). Similarly, a systematic review and meta-analysis by Xu et al assessing outcomes in acute hypoxemic, nonhypercapnic respiratory failure found that helmet NIV, but not nasal mask or facemask NIV, could reduce hospital mortality (17). This data suggests that helmet NIV should be utilized over facemask NIV when both modalities are available. Apart from outcomes, helmet NIV has several practical advantages to facemask NIV. Helmet NIV tends to be more comfortable and better tolerated by patients compared to facemask NIV, as helmet NIV utilizes a soft airtight collar around the neck rather than the tight seal around the face utilized in facemask NIV. Helmet NIV may also allow clinicians to use higher pressures compared to facemask NIV, as high pressures in facemask NIV often result in air leak. Helmet NIV may also help reduce pressure sores associated with facemask NIV as the primary point of contact is around the neck rather than over the face. BaHamman et al reviewed the different interfaces available to clinicians for delivery of NIV, including nasal mask, oral mask, oro-nasal mask, nasal pillows, face mask and helmet. Compared to other interfaces, helmet NIV is superior in that it allows patients to communicate, expectorate, and even drink fluids without interrupting the therapy (18). Helmet NIV may also help reduce the incidence of common complications associated with other NIV interfaces including air leak, nasal congestion, oro-nasal dryness, skin irritation, eye irritation, and claustrophobia (18).

Therefore, when considering NIV, evidence suggests that helmet NIV should be the modality of choice given an option between helmet and facemask. Realistically, however, the determining factor is often which type(s) of NIV each hospital is equipped with. For hospitals with both modalities available, helmet appears superior and should be the first choice, whereas clinicians without access to both modalities should use the mode of NIV available to them. For patients on a trial of face mask NIV, there is currently no role in converting from facemask to helmet NIV if deteriorating on facemask NIV; rather, the next best step would be mechanical ventilation as there simply isn't enough data to support delaying intubation in a patient deteriorating on facemask NIV for a trial of helmet NIV. It is quite clear that the type of NIV provided, the delivery method, and the timing of NIV onset all play a role in patient outcomes.

A recently published review article by Grieco et al. outlines the role of NIRS in the management of hypoxemic respiratory failure and ARDS. Grieco et al. postulate that NIRS can be utilized as first line treatment so long as efforts are made to prevent the development of patient self-inflicted lung injury, which may be prevented with high PEEP (10-15 cm H2O), pharmacologic agents such as propofol and benzodiazepines, and a focus on management of complicating factors such as pain, fever, and metabolic acidosis (19). HFNC is suggested as first line treatment, with reservation of high-PEEP helmet NIV for patients with more severe disease that do not yet meet criteria for IMV (19). Grieco et al. outline several physiological measures that should be monitored during NIRS including SpO_2_/FiO_2_, PaO_2_/FiO_2_, respiratory rate, expired tidal volume, change in esophageal pressure, ROX index, and the HACOR scale (heart rate, acidosis, consciousness, oxygenation, and respiratory rate) (19).

*Middle East Respiratory Syndrome Coronavirus*

The 2012 outbreak of Middle East Respiratory syndrome coronavirus (MERS) resulted in acute hypoxemic respiratory failure, multi-organ dysfunction, and high mortality (20). In an analysis of critically ill patients with MERS, 35% were initially treated with NIV and 65% with IMV (21). Patients managed with NIV had a lower SOFA score, less extensive infiltrates on chest radiograph, and higher Glasgow Coma Score (21). An overwhelming 92.4% of patients initially managed with NIV ultimately required IMV and were more likely to need inhaled nitric oxide compared to those immediately intubated; however, ICU length of stay, hospital length of stay and 90-day mortality were similar between groups (21). Subsequent subgroup analysis found, when compared to the NIV failure group, the NIV success group was significantly younger, had lower SOFA scores, and had lower 90-day mortality (21).

*Severe Acute Respiratory Syndrome Coronavirus (SARS)*

During the 2003 SARS epidemic, patients presented in varying degrees of respiratory distress with approximately 20% of patients requiring critical care for ARF (22). The mortality rate for individuals diagnosed with SARS who required intensive care was 34-53% at 28 days (22). NIV use was found to be effective in the management of SARS, however, the incidence of NIV‐associated barotrauma was 6.6-15% (22). Cheung et al reported the use of NIV in patients with ARF secondary to SARS and found that NIV resulted in the avoidance of IMV 70% of the time and was associated with shorter ICU length of stay (23). IMV avoidance was predicted by a reduction in both respiratory rate and supplemental oxygen requirement within 24 hours of initiating NIV (23). None of the HCW who cared for patients receiving NIV developed SARS (23). Similarly, Yam et al compared NIV to IMV in SARS patients and found a reduction in intubation rate and mortality with no transmission of SARS to HCW with the use of NIV (24).

*H1N1*

NIV was also deployed as a treatment strategy during the 2009 H1N1 pandemic. Rello et al studied NIV use in patients with acute respiratory failure secondary to H1N1 and found 75% failed NIV and required IMV (25). A separate study of ICU patients with H1N1 reported that NIV was used in 19% of cases and was associated with decreased mortality (26). In a subgroup analysis, 24% of survivors were found to have received a trial of NIV prior to requiring IMV, whereas only 13% of nonsurvivors received NIV prior to IMV (26). A study by Masclans et al found that NIV in H1N1 patients was successful 40.7% of the time (27). NIV success patients had shorter hospital stays and lower mortality rates, similar to those of non-ventilated patients. Importantly, the mortality rate of patients in whom NIV failed was similar to that of patients who required immediate intubation (27). Lower APACHE II score, lower SOFA score, hemodynamic stability defined as the absence of vasopressors, and the absence of multi-organ dysfunction were associated with NIV success (27). Similarly, Rodriguez et al studied patients with ARF secondary to influenza and found that 56.8% failed NIV; APACHE II score, SOFA score, infiltrates on imaging, and ICU mortality were higher in the NIV failure group compared to the NIV success group. Specifically, a SOFA score ≥ 5 was associated with NIV failure (28). When compared to the group who was immediately intubated, ICU mortality was higher in the NIV failure group (28).

**References**

1. Confalonieri M, Potena A, Carbone G, Porta RD, Tolley EA, Umberto Meduri G. Acute respiratory failure in patients with severe community-acquired pneumonia. A prospective randomized evaluation of noninvasive ventilation. Am J Respir Crit Care Med. 1999;160(5 Pt 1):1585-91.

2. Egea N, Cazaux A, Langer M, Cambursano H. Noninvasive Positive-Pressure Ventilation in Patients with Acute Hypoxemic Respiratory Failure and HIV/AIDS. In: Esquinas AM, editor. Noninvasive Ventilation in High-Risk Infections and Mass Casualty Events. Vienna: Springer Vienna; 2014. p. 85-97.

3. David-Joao PG, Guedes MH, Rea-Neto A, Chaiben VBO, Baena CP. Noninvasive ventilation in acute hypoxemic respiratory failure: A systematic review and meta-analysis. J Crit Care. 2019;49:84-91.

4. Klefti G, Hill AT. The benefits of non-invasive ventilation for Community-Acquired Pneumonia: A meta-analysis. QJM. 2020.

5. Bellani G, Laffey JG, Pham T, Madotto F, Fan E, Brochard L, et al. Noninvasive Ventilation of Patients with Acute Respiratory Distress Syndrome. Insights from the LUNG SAFE Study. Am J Respir Crit Care Med. 2017;195(1):67-77.

6. Antonelli M, Conti G, Esquinas A, Montini L, Maggiore SM, Bello G, et al. A multiple-center survey on the use in clinical practice of noninvasive ventilation as a first-line intervention for acute respiratory distress syndrome. Crit Care Med. 2007;35(1):18-25.

7. Tonelli R, Fantini R, Tabbi L, Castaniere I, Pisani L, Pellegrino MR, et al. Early Inspiratory Effort Assessment by Esophageal Manometry Predicts Noninvasive Ventilation Outcome in De Novo Respiratory Failure. A Pilot Study. Am J Respir Crit Care Med. 2020;202(4):558-67.

8. Cabrini L, Landoni G, Oriani A, Plumari VP, Nobile L, Greco M, et al. Noninvasive ventilation and survival in acute care settings: a comprehensive systematic review and metaanalysis of randomized controlled trials. Crit Care Med. 2015;43(4):880-8.

9. Agarwal R, Reddy C, Aggarwal AN, Gupta D. Is there a role for noninvasive ventilation in acute respiratory distress syndrome? A meta-analysis. Respir Med. 2006;100(12):2235-8.

10. Lenglet H, Sztrymf B, Leroy C, Brun P, Dreyfuss D, Ricard JD. Humidified high flow nasal oxygen during respiratory failure in the emergency department: feasibility and efficacy. Respir Care. 2012;57(11):1873-8.

11. Frat JP, Thille AW, Mercat A, Girault C, Ragot S, Perbet S, et al. High-flow oxygen through nasal cannula in acute hypoxemic respiratory failure. N Engl J Med. 2015;372(23):2185-96.

12. Grieco DL, Menga LS, Raggi V, Bongiovanni F, Anzellotti GM, Tanzarella ES, et al. Physiological Comparison of High-Flow Nasal Cannula and Helmet Noninvasive Ventilation in Acute Hypoxemic Respiratory Failure. Am J Respir Crit Care Med. 2020;201(3):303-12.

13. Ferreyro BL, Angriman F, Munshi L, Del Sorbo L, Ferguson ND, Rochwerg B, et al. Association of Noninvasive Oxygenation Strategies With All-Cause Mortality in Adults With Acute Hypoxemic Respiratory Failure: A Systematic Review and Meta-analysis. JAMA. 2020;324(1):57-67.

14. Patel BK, Wolfe KS, Pohlman AS, Hall JB, Kress JP. Effect of Noninvasive Ventilation Delivered by Helmet vs Face Mask on the Rate of Endotracheal Intubation in Patients With Acute Respiratory Distress Syndrome: A Randomized Clinical Trial. JAMA. 2016;315(22):2435-41.

15. Chaudhuri D, Jinah R, Burns KEA, Angriman F, Ferreyro B, Munshi L, et al. Helmet non-invasive ventilation compared to facemask non-invasive ventilation and high flow nasal cannula in acute respiratory failure: a systematic review and meta-analysis. Eur Respir J. 2021.

16. Liu Q, Gao Y, Chen R, Cheng Z. Noninvasive ventilation with helmet versus control strategy in patients with acute respiratory failure: a systematic review and meta-analysis of controlled studies. Crit Care. 2016;20:265.

17. Xu XP, Zhang XC, Hu SL, Xu JY, Xie JF, Liu SQ, et al. Noninvasive Ventilation in Acute Hypoxemic Nonhypercapnic Respiratory Failure: A Systematic Review and Meta-Analysis. Crit Care Med. 2017;45(7):e727-e33.

18. BaHammam AS, Singh TD, Gupta R, Pandi-Perumal SR. Choosing the Proper Interface for Positive Airway Pressure Therapy in Subjects With Acute Respiratory Failure. Respir Care. 2018;63(2):227-37.

19. Grieco DL, Maggiore SM, Roca O, Spinelli E, Patel BK, Thille AW, et al. Non-invasive ventilatory support and high-flow nasal oxygen as first-line treatment of acute hypoxemic respiratory failure and ARDS. Intensive Care Med. 2021;47(8):851-66.

20. Arabi YM, Arifi AA, Balkhy HH, Najm H, Aldawood AS, Ghabashi A, et al. Clinical course and outcomes of critically ill patients with Middle East respiratory syndrome coronavirus infection. Ann Intern Med. 2014;160(6):389-97.

21. Alraddadi BM, Qushmaq I, Al-Hameed FM, Mandourah Y, Almekhlafi GA, Jose J, et al. Noninvasive ventilation in critically ill patients with the Middle East respiratory syndrome. Influenza Other Respir Viruses. 2019;13(4):382-90.

22. Yam LY, Chen RC, Zhong NS. SARS: ventilatory and intensive care. Respirology. 2003;8 Suppl:S31-5.

23. Cheung TM, Yam LY, So LK, Lau AC, Poon E, Kong BM, et al. Effectiveness of noninvasive positive pressure ventilation in the treatment of acute respiratory failure in severe acute respiratory syndrome. Chest. 2004;126(3):845-50.

24. Yam LY, Chan AY, Cheung TM, Tsui EL, Chan JC, Wong VC, et al. Non-invasive versus invasive mechanical ventilation for respiratory failure in severe acute respiratory syndrome. Chin Med J (Engl). 2005;118(17):1413-21.

25. Rello J, Rodriguez A, Ibanez P, Socias L, Cebrian J, Marques A, et al. Intensive care adult patients with severe respiratory failure caused by Influenza A (H1N1)v in Spain. Crit Care. 2009;13(5):R148.

26. Estenssoro E, Rios FG, Apezteguia C, Reina R, Neira J, Ceraso DH, et al. Pandemic 2009 influenza A in Argentina: a study of 337 patients on mechanical ventilation. Am J Respir Crit Care Med. 2010;182(1):41-8.

27. Masclans JR, Perez M, Almirall J, Lorente L, Marques A, Socias L, et al. Early non-invasive ventilation treatment for severe influenza pneumonia. Clin Microbiol Infect. 2013;19(3):249-56.

28. Rodriguez A, Ferri C, Martin-Loeches I, Diaz E, Masclans JR, Gordo F, et al. Risk Factors for Noninvasive Ventilation Failure in Critically Ill Subjects With Confirmed Influenza Infection. Respir Care. 2017;62(10):1307-15.
