# Supplementary material for: Noninvasive respiratory support for COVID-19 patients: when, for whom, and how?
Source: J Intensive Care. 2022 Jan 15;10:3. doi: 10.1186/s40560-021-00593-1 (PMC8760575; doi:10.1186/s40560-021-00593-1)
Supplement: Supplementary file 3 — Additional file 3: Text S3. Prone positioning. [file 40560_2021_593_MOESM3_ESM.docx]

***Prone Positioning***

One of the strategies utilized in the fight against COVID-19 that has potential widespread implications for future management is awake prone positioning, both with and without concurrent use of NIRS. The current evidence for prone positioning without NIRS in COVID-19 patients is conflicting. A prospective cohort study by Zang et al found significant improvement in SpO_2_, RR, ROX index, and reduced mortality in COVID-19 patients with severe hypoxia treated with supplemental oxygen and early prone positioning compared to patients on supplemental oxygen alone^1^. Similarly, the POSITIONED study by Jagan et al found awake self-proning in COVID-19 patients to be associated with decreased mortality and intubation rates^2^. However, a retrospective cohort study by Padrão et al evaluating patients with suspected COVID-19 on supplemental oxygen showed no significant difference in rates of intubation between the awake prone positioning group and the non-prone group^3^. A recent systematic review by Pavlov et al. comparing intubation rates between COVID-19 patients treated with awake prone positioning versus standard therapy found that awake prone positioning was associated with improvement in oxygenation, but had no effect on intubation rate^4^.

Prone positioning has also been studied in patients who are concurrently trialing NIRS. Ding et al evaluated the efficacy of early prone positioning combined with either HFNC or NIV in preventing intubation in patients with moderate-severe ARDS^5^. The four groups in the study were HFNC, HFNC + prone, NIV, and NIV + prone^5^. Investigators concluded that early prone in combination with either NIV or HFNC may reduce the intubation rate in patients with moderate ARDS with SpO_2_ >95%, and that patients with severe ARDS were poor candidates for attempted NIV + prone or HFNC + prone^5^. Importantly, investigators identified the largest increase in PaO_2_/FIO_2_ to be in the NIV + prone group, followed by the NIV group, then HFNC + prone group, and lastly, the HFNC group^5^. Xu et al expanded on these findings with a retrospective observational case series evaluating outcomes in COVID-19 patients with ARDS (defined as PaO_2_/FiO_2_ <300) treated with early prone positioning and HFNC^6^. The case series evaluated 10 patients with severe COVID-19 ARDS (PaO_2_/FiO_2_ ratio ranged from 89 to 228) who received HFNC and early proning and found that the PaO_2_/FiO_2_ ratio was significantly improved following proning and all 10 subjects avoided IMV^6^. In contrast, a prospective, multicenter, adjusted observational cohort study conducted by Ferrando et al assessing awake prone positioning in COVID-19 patients on HFNC showed no reduction in intubation rates or mortality in the prone group^7^.

Retucci et al conducted a prospective, observational study of COVID-19 patients to assess the efficacy of a one hour trial of helmet CPAP in the prone and lateral position with success defined as a decrease in the PAO_2_-PaO_2_ gradient of at least 20%, unchanged or improved tachypnea, and unchanged or improved dyspnea^8^. Prone positioning was used in patients with bilateral lung involvement with a success rate of 33.3% and lateral positioning was used in patients with single lung involvement with a success rate of 8%^8^. Unfortunately, the improvement in air exchange obtained in the prone and lateral position was lost once patients transitioned back to a semi-seated position^8^. Sartini et al. evaluated the efficacy of NIV in the prone position in patients with COVID-19 and found that, while prone, patients had improved oxygenation, increased PaO_2_/FiO_2_ ratio, and decreased respiratory rate^9^. Longhini et al have proposed helmet CPAP and prone positioning as an early treatment strategy for COVID-19 with inclusion criteria being PaO_2_/FiO_2_ 200-300, SpO_2_ <95% on room air, and dyspnea, with exclusion criteria being COPD, PaCO_2_ > 50, and pregnancy^10^.

Prone trial length is often limited by patient tolerance and comorbid conditions. Therefore, the length of a prone trial is largely patient specific but should last at least one hour if the patient does not deteriorate to the point of requiring mechanical ventilation. If the patient improves in the prone position, it is best to keep them in that position for as long as tolerated, as the gains made in the prone position may be lost once they return to the supine position. Guerin et al conducted a prospective RCT evaluating the effect of prone positioning on intubated patients with severe ARDS and found that early prone positioning of at least 16 hours was associated with significantly decreased 28-day and 90-day mortality when compared to the supine group ^11^. These results suggest that at least 16 hours would be the ideal length for a prone trial, however the patients in this study were intubated and sedated, making a prolonged prone trial much easier to tolerate when compared to a prone trial in awake patients on NIRS. Therefore, the ideal length of a prone trial in awake patients on NIRS would be greater than 1 hour and up to 16 hours if tolerated well by the patient. However, the Society of Critical Care Medicine (SCCM) recently released updated recommendations for the management of COVID-19 and provided no recommendation for prone positioning in nonintubated patients due to limited clinical evidence^12^.

**References**

1. Zang X, Wang Q, Zhou H, Liu S, Xue X and Group C-EPPS. Efficacy of early prone position for COVID-19 patients with severe hypoxia: a single-center prospective cohort study. *Intensive Care Med*. 2020;46:1927-1929.

2. Jagan N, Morrow LE, Walters RW, Klein LP, Wallen TJ, Chung J and Plambeck RW. The POSITIONED Study: Prone Positioning in Nonventilated Coronavirus Disease 2019 Patients-A Retrospective Analysis. *Crit Care Explor*. 2020;2:e0229.

3. Padrao EMH, Valente FS, Besen B, Rahhal H, Mesquita PS, de Alencar JCG, da Costa MGP, Wanderley APB, Emerenciano DL, Bortoleto FM, Fortes JCL, Marques B, de Souza SFB, Marchini JFM, Neto RAB, de Souza HP and Covidteam. Awake Prone Positioning in COVID-19 Hypoxemic Respiratory Failure: Exploratory Findings in a Single-center Retrospective Cohort Study. *Acad Emerg Med*. 2020;27:1249-1259.

4. Pavlov I, He H, McNicholas B, Perez Y, Tavernier E, Trump MW, Jackson JA, Zhang W, Rubin DS, Spiegel T, Hung A, Estrada MAI, Roca O, Vines DL, Cosgrave D, Mirza S, Laffey JG, Rice TW, Ehrmann S and Li J. Awake prone positioning in non-intubated patients with acute hypoxemic respiratory failure due to COVID-19: A systematic review of proportional outcomes comparing observational studies with and without awake prone positioning in the setting of COVID-19. *Respir Care*. 2021.

5. Ding L, Wang L, Ma W and He H. Efficacy and safety of early prone positioning combined with HFNC or NIV in moderate to severe ARDS: a multi-center prospective cohort study. *Crit Care*. 2020;24:28.

6. Xu Q, Wang T, Qin X, Jie Y, Zha L and Lu W. Early awake prone position combined with high-flow nasal oxygen therapy in severe COVID-19: a case series. *Crit Care*. 2020;24:250.

7. Ferrando C, Mellado-Artigas R, Gea A, Arruti E, Aldecoa C, Adalia R, Ramasco F, Monedero P, Maseda E, Tamayo G, Hernandez-Sanz ML, Mercadal J, Martin-Grande A, Kacmarek RM, Villar J, Suarez-Sipmann F and Network C-SI. Awake prone positioning does not reduce the risk of intubation in COVID-19 treated with high-flow nasal oxygen therapy: a multicenter, adjusted cohort study. *Crit Care*. 2020;24:597.

8. Retucci M, Aliberti S, Ceruti C, Santambrogio M, Tammaro S, Cuccarini F, Carai C, Grasselli G, Oneta AM, Saderi L, Sotgiu G, Privitera E and Blasi F. Prone and Lateral Positioning in Spontaneously Breathing Patients With COVID-19 Pneumonia Undergoing Noninvasive Helmet CPAP Treatment. *Chest*. 2020;158:2431-2435.

9. Sartini C, Tresoldi M, Scarpellini P, Tettamanti A, Carco F, Landoni G and Zangrillo A. Respiratory Parameters in Patients With COVID-19 After Using Noninvasive Ventilation in the Prone Position Outside the Intensive Care Unit. *JAMA*. 2020;323:2338-2340.

10. Longhini F, Bruni A, Garofalo E, Navalesi P, Grasselli G, Cosentini R, Foti G, Mattei A, Ippolito M, Accurso G, Vitale F, Cortegiani A and Gregoretti C. Helmet continuous positive airway pressure and prone positioning: A proposal for an early management of COVID-19 patients. *Pulmonology*. 2020;26:186-191.

11. Guerin C, Reignier J, Richard JC, Beuret P, Gacouin A, Boulain T, Mercier E, Badet M, Mercat A, Baudin O, Clavel M, Chatellier D, Jaber S, Rosselli S, Mancebo J, Sirodot M, Hilbert G, Bengler C, Richecoeur J, Gainnier M, Bayle F, Bourdin G, Leray V, Girard R, Baboi L, Ayzac L and Group PS. Prone positioning in severe acute respiratory distress syndrome. *N Engl J Med*. 2013;368:2159-68.

12. Alhazzani W, Evans L, Alshamsi F, Moller MH, Ostermann M, Prescott HC, Arabi YM, Loeb M, Ng Gong M, Fan E, Oczkowski S, Levy MM, Derde L, Dzierba A, Du B, Machado F, Wunsch H, Crowther M, Cecconi M, Koh Y, Burry L, Chertow DS, Szczeklik W, Belley-Cote E, Greco M, Bala M, Zarychanski R, Kesecioglu J, McGeer A, Mermel L, Mammen MJ, Nainan Myatra S, Arrington A, Kleinpell R, Citerio G, Lewis K, Bridges E, Memish ZA, Hammond N, Hayden FG, Alshahrani M, Al Duhailib Z, Martin GS, Kaplan LJ, Coopersmith CM, Antonelli M and Rhodes A. Surviving Sepsis Campaign Guidelines on the Management of Adults With Coronavirus Disease 2019 (COVID-19) in the ICU: First Update. *Crit Care Med*. 2021;49:e219-e234.
